# Occupational Exposure and Dementia Risk in the Swedish Adoption/Twin Study of Aging

**DOI:** 10.1002/alz70860_106104

**Published:** 2025-12-23

**Authors:** Otto‐Emil Ilmari Jutila, Michelle Luciano, Tom C Russ, Ida K Karlsson

**Affiliations:** ^1^ University of Edinburgh, Edinburgh, United Kingdom; ^2^ Lothian Birth Cohort studies, Edinburgh, United Kingdom; ^3^ Alzheimer Scotland Dementia Research Centre, University of Edinburgh, Edinburgh, United Kingdom; ^4^ University of Edinburgh, Edinburgh, Scotland, United Kingdom; ^5^ Centre for Dementia Prevention, University of Edinburgh, Edinburgh, United Kingdom; ^6^ Centre for Clinical Brain Sciences, University of Edinburgh, Edinburgh, United Kingdom; ^7^ Karolinska Institutet, Stockholm, Sweden

## Abstract

**Background:**

Occupational exposure to inhalable airborne toxicants has been associated with later life dementia but the findings are inconsistent, and research is limited. The mechanism has been uncertain, with genetic and environmental influences are difficult to disentangle. This study examines the relationship between occupational exposures and dementia risk in a twin sample facilitating assessment of familial confounding.

**Method:**

Data were obtained from 316 participants (50.32% women) in the Swedish Adoption/Twin Study on Aging (SATSA), a longitudinal twin cohort. Inhalable occupational exposures (dust, soot, and/or gases; smoke; odours; damp air; solvents; insufficient ventilation; and dirt) were self‐reported in the first questionnaire during1984 (mean age=51.57 years). Principal component analysis was applied to occupational exposures to derive the first principal component (PC1), representing a multipollutant occupational exposure. All‐cause dementia diagnoses (*N* = 48) were based on cognitive testing, clinically evaluation, and healthcare register linkage until 2016. Cox proportional hazards models were used to examine the association between PC1 and individual occupational risk factors with all‐cause dementia. Additional within‐twin pair analyses were conducted using stratified Cox models, to account for genetic and other familial influences. Models were adjusted by age, sex, education, and smoking.

**Result:**

Occupational exposure (PC1) was not associated with risk of dementia in the total sample (HR 1.05, 95% CI 0.87‐1.26). Exposure to dust, soot, and/or gases was associated with higher risk of dementia in the total sample (HR 1.99, 95% CI 1.06‐3.74), with a comparable estimate in within‐twin pair analyses (HR 2.30, 95% CI 0.31‐17.29). Associations between other occupational exposures and dementia were not statistically significant: smoke (HR,1.23, 95% CI 0.62‐2.44), smell (HR, 0.86, 95% CI 0.44‐1.68), damp air (HR, 0.87, 95% CI 0.38‐2.00), solvents (HR, 0.62, 95% CI 0.26‐1.47), insufficient ventilation (HR, 0.96, 95% CI 0.50‐1.82), and dirt (HR, 1.60, 95% CI 0.83‐3.10).

**Conclusion:**

Exposure to dust, soot, and gases may be associated with higher risk of dementia, with comparable effect estimates within twin pairs indicating that the association is not explained by familial confounding. No statistically significant associations were between other inhalable occupational exposures and dementia risk. Future research should involve larger cohorts with more detailed occupational exposure measurements.

